# Associations between Tobacco, Alcohol, and Drug Use with Coronary Artery Plaque among HIV-Infected and Uninfected Men in the Multicenter AIDS Cohort Study

**DOI:** 10.1371/journal.pone.0147822

**Published:** 2016-01-26

**Authors:** Sean G. Kelly, Michael Plankey, Wendy S. Post, Xiuhong Li, Ronald Stall, Lisa P. Jacobson, Mallory D. Witt, Lawrence Kingsley, Christopher Cox, Matthew Budoff, Frank J. Palella

**Affiliations:** 1 Northwestern University Feinberg School of Medicine, Chicago, Illinois, United States of America; 2 Georgetown University, Washington, D. C., United States of America; 3 Johns Hopkins University School of Medicine, Baltimore, Maryland, United States of America; 4 Johns Hopkins Bloomberg School of Public Health, Baltimore, Maryland, United States of America; 5 University of Pittsburgh School of Public Health, Pittsburgh, Pennsylvania, United States of America; 6 Harbor-UCLA Medical Center, Torrance, California‎, United States of America; University of Pittsburgh Center for Vaccine Research, UNITED STATES

## Abstract

**Background:**

We characterized associations between smoking, alcohol, and recreational drug use and coronary plaque by HIV serostatus within the Multicenter AIDS Cohort Study (MACS).

**Methods:**

MACS participants (N = 1005, 621 HIV+ and 384 HIV-) underwent non-contrast CT scanning to measure coronary artery calcium; 764 underwent coronary CT angiograms to evaluate plaque type and extent. Self-reported use of alcohol, tobacco, smoked/inhaled cocaine, methamphetamine, ecstasy, marijuana, inhaled nitrites, and erectile dysfunction drugs was obtained at semi-annual visits beginning 10 years prior to CT scanning. Multivariable logistic and linear regression models were performed, stratified by HIV serostatus.

**Results:**

Among HIV+ men, current smoking, former smoking, and cumulative pack years of smoking were positively associated with multiple coronary plaque measures (coronary artery calcium presence and extent, total plaque presence and extent, calcified plaque presence, and stenosis >50%). Smoking was significantly associated with fewer plaque measures of comparable effect size among HIV- men; current smoking and calcified plaque extent was the only such association. Heavy alcohol use (>14 drinks/week) was associated with stenosis >50% among HIV+ men. Among HIV- men, low/moderate (1–14 drinks/week) and heavy alcohol use were inversely associated with coronary artery calcium and calcified plaque extent. Few significant associations between other recreational drug use and plaque measures were observed.

**Conclusion:**

Smoking is strongly associated with coronary plaque among HIV+ men, underscoring the value of smoking cessation for HIV+ persons. Alcohol use may protect against coronary artery calcium and calcified plaque progression in HIV- (but not HIV+) men. Few positive associations were observed between recreational drug use and coronary plaque measures.

## Introduction

As survival among human immunodeficiency virus (HIV)-infected persons has been markedly extended through the routine use of potent combination antiretroviral therapy (cART) [1], non-infectious chronic diseases associated with aging in the general population are more prevalent. Multiple studies suggest an increased risk of coronary vascular disease (CVD) among HIV+ persons compared to the general population despite cART-associated HIV viral suppression [2–5]. Factors implicated in increased CVD risk among HIV+ persons include chronic immune activation and inflammation (possibly due to chronic low level viremia), specific ART drug exposure, and increased prevalence of traditional CVD risk factors [2,3,6–10]. HIV+ persons have an increased prevalence of coronary artery calcium compared to HIV- persons [5,6,11] and a recent report from our group that evaluated subclinical plaque using CT angiography demonstrated higher non-calcified plaque prevalence and extent in HIV+ men compared to HIV- men [12]. Non-calcified plaque, which is lipid-rich, strongly pro-inflammatory, prone to rupture and thrombosis, and carries a high risk of major coronary events, has previously been associated with HIV infection using CT angiography [13,14]. Associations between HIV infection and other plaque types (calcified plaque and mixed plaque, which contains <50% calcification), which are thought to be more stable and portend lower risk of fatal coronary events than non-calcified plaque [13], are less distinct.

Smoking, alcohol and recreational drug use contribute to CVD development, ranging from subclinical atherosclerosis to fatal acute coronary syndromes (ACS) [15–20]. Nationally, HIV+ persons are more likely to abuse alcohol and use recreational drugs (notably marijuana, cocaine and other stimulants) than HIV- persons [21,22]. The detrimental effect of cocaine use on coronary disease pathogenesis has been demonstrated [23,24], and a synergistic effect of cocaine use and HIV infection on endothelial dysfunction and coronary atherosclerosis has been suggested [25,26]. Otherwise, few data exist regarding specific substance use and CVD risk in the HIV+ population.

The extent to which smoking, alcohol use and recreational drug use among HIV+ persons affects CVD risk compared to such use in HIV- persons has not been well-characterized. We performed a retrospective analysis of prospectively collected data from Multicenter AIDS Cohort Study (MACS) participants (including previously reported upon coronary CT angiography [CTA] data) to investigate associations of alcohol consumption, tobacco use and recreational drug use with the presence, extent and type of subclinical coronary artery plaque among HIV+ and HIV- men.

## Methods

The MACS is an ongoing, prospective cohort study evaluating the natural history of treated and untreated HIV infection [27]. Both HIV+ and HIV- men who have sex with men (MSM) undergo semiannual standardized interviews, physical examinations and storage and analyses of blood and urine specimens. Cohort enrollment began in 1984–1985 with further enrichment in 1987–1991 and in 2001–2003 [27,28].

This analysis included data from participants enrolled in an ancillary cardiovascular study. Written consent was obtained from all participants, and the study was approved by the institutional review boards at all participating sites (Johns Hopkins Medicine Institutional Review Board, Baltimore, MD; University of Pittsburgh Institutional Review Board, Pittsburgh, PA; Northwestern University Institutional Review Board, Chicago, IL; University of California, Los Angeles Institutional Review Board, Los Angeles, CA). The study included men age 40–70 years, weighing <300 lbs. with no prior history of cardiac surgery or percutaneous coronary artery intervention. All participants underwent non-contrast cardiac CT scanning for coronary artery calcium scoring (Agatston score). Men eligible for CTA (absence of atrial fibrillation, estimated glomerular filtration rate [eGFR] >60 mL/min/m^2^, absence of history of intravenous contrast allergy) also underwent CTA scanning to evaluate coronary artery plaque type and extent [12].

### Participant Clinical Parameters

Data regarding substance use and HIV clinical parameters were obtained prospectively from participants by self-reported history, physical exam and laboratory analysis of blood specimens collected at each semiannual MACS visit and generally within six months prior to CT scanning. Serum glucose, total, high-density lipoprotein cholesterol and triglyceride levels were measured from fasting blood samples. Low-density lipoprotein was calculated with the Friedewald equation, or measured directly in nonfasting samples or in the presence of triglycerides >400 mg/dL. Serum creatinine was measured at each MACS visit, and within 30 days prior to CT scanning for men eligible for IV contrast injection. GFR was calculated using the Modification of Diet in Renal Disease equation (MDRD). Diabetes mellitus was defined as fasting serum glucose >126 mg/dL or the reported use of diabetic medications. Hypertension was defined as systolic blood pressure (BP) >140 or diastolic BP >90 mmHg, or reported use of antihypertensive medications. In HIV+ men, plasma HIV RNA levels, CD4+ T-lymphocyte (CD4) nadir, and history of AIDS-defining illness were included as measures of HIV disease status. In the MACS, plasma HIV RNA was measured using the COBAS Ultrasensitive Amplicor HIV-1 monitor assay (Roche Molecular Systems, Branchburg, NJ), sensitive to 50 copies HIV RNA/mL, or Taqman HIV-1 Test (Roche molecular Systems, Branchburg, NJ), sensitive to 20 copies HIV RNA/mL. CD4 counts were measured using standardized flow cytometry [29]. AIDS-defining illnesses were identified (or determined) according to the 1993 CDC definition of AIDS [30]. Body mass index (BMI) was determined at each visit by dividing measured body weight (kg) by measured height^2^ (m^2^). For this analysis, we used clinical data and blood tests that had been collected at the previous MACS visit closest to the CT scan, generally within 6 months [12].

### Substance Use

Substance use was the primary exposure of interest, defined as alcohol consumption, use of smoked tobacco or recreational drugs. Recreational drug use included stimulants (smoked or inhaled cocaine, methamphetamines, and ecstasy), marijuana, inhaled nitrites, and erectile dysfunction drugs (EDD; phosphodiesterase inhibitors, including both prescribed medications and non-prescribed medications used recreationally for sexual performance enhancement). EDD were included due to the high prevalence of their use in our cohort and the association with comorbid use of other recreational substances among MSM [31]. Further, the vasoactive properties of EDD, increasing nitric oxide production and thus facilitating coronary vasodilation, entrench their inclusion in this analysis. Self-reported use of other substances was obtained prospectively from semiannual questionnaires performed during the 10-year period prior to CT scanning. For smoking, questionnaires asked if the participants had ever smoked cigarettes, if they currently smoke cigarettes, and how many packs they smoke daily. For alcohol consumption, questionnaires asked the participants how frequently they drank alcohol during the previous six months (never, less than monthly, monthly, weekly, daily or almost daily), and how many drinks they consumed on a typical day when they were drinking. For other recreational drug use, questionnaires asked the participants if they had used the substance in question since the previous MACS visit, and if so, how frequently they used the substance (daily, weekly, monthly, less often).

#### Alcohol

Use patterns in the 6 months before CT scanning were categorized as none (reference group), low/moderate use (1–14 drinks/week), and heavy use (>14 drinks/week). Binge drinking (≥5 drinks at least once over past 30 days) was included as a separate, distinct variable.

#### Tobacco

Smoking was categorized as no use (reference group), current use, or former use at time of CT scanning, and cumulative pack-years before and up to CT scanning (lifetime pack-years were analyzed rather than only including the 10-year study period).

#### Recreational drugs

These use patterns were defined as cumulative years of the substance used, weighted by frequency of use, during the 10-year study period. Similar to the standard measurement of smoking in pack-years (packs per day, per year), we determined use-years for each of the other recreational substances. Since recreational substances were likely to be used less frequently than tobacco, use-years for recreational drugs were standardized to monthly use (as opposed to daily use as with tobacco smoking). Thus, use-years were characterized as use of drug per month, per year. For example, if an individual reported daily use of a substance during the last six months, then his use was calculated as 30.5 days per month x 0.5 years = 15.25 years of monthly use (or 15.25 use-years). Alternatively, if the individual used the same substance once monthly, then his use was calculated as 1 day per month x 0.5 years = 0.5 use-years (similar conceptually to pack-years of smoking; if the individual smoked 1 pack daily over six months, his calculated average use is 0.5 pack-years). Using this method of calculating use-years at each semiannual study visit, cumulative use over the 10-year study period was determined (for further discussion on cumulative use of recreational drugs methodology, see Appendix).

### CT Scanning

Procedural details of the cardiac CT scanning have been previously described [12,32]. Briefly, three centers utilized a 64-slice multi-detector CT and one center utilized a 320-row multi-detector CT. Participants received a beta-blocker or calcium channel blocker as needed for heart rate control, and sublingual nitroglycerine prior to IV contrast injection unless contraindicated.

CT and CTA images were analyzed by experienced readers blinded to participant HIV serostatus at the Los Angeles Biomedical Research Institute at Harbor-UCLA. Coronary artery segments were analyzed using the 15-segment model of the American Heart Association [33]. The presence, extent and calcium composition of coronary artery plaques, as well as degree of luminal narrowing, were assessed in all measurable segments. Plaque size was graded as 0 = no plaque, 1 = mild, 2 = moderate, or 3 = severe. Segment stenosis was defined as 0 = no plaque, 1 = 1–29% (minimal) stenosis, 2 = 30–49% (mild) stenosis, 3 = 50–69% (moderate) stenosis, or 4 for ≥70% (severe) stenosis. The total plaque score was calculated by summing the plaque size score for all assessable coronary segments that demonstrated any plaque (either calcified, non-calcified, or mixed) up to a maximum score of 45. Plaques were classified as calcified (>130 Hounsfield Units visualized as separate from the vessel lumen in at least two independent planes), non-calcified (discernible intraluminal structures assigned to a vessel wall with CT density less than that of the intravascular contrast but greater than that of the surrounding connective tissue), and mixed (<50% of the plaque occupied by calcium). Total plaque scores were produced by the summation of plaque scores in each calcified, non-calcified, and mixed plaque segments separately.

### Statistical Analysis

Since factors associated with plaque presence (present vs. absent) and extent (plaque scores greater than zero) may differ, they were treated as separate outcomes. Separate multiple logistic regression models were performed to evaluate associations between substance use and plaque presence for coronary artery calcium, total plaque, calcified plaque, non-calcified plaque, mixed plaque, and coronary artery stenosis >50%. Those with coronary artery calcium or plaque presence (that is to say, presence of subclinical atherosclerosis as determined by coronary artery calcium, total plaque, calcified plaque, non-calcified plaque, and mixed plaque scores above zero), underwent further analysis by linear regression to evaluation associations between substance use and plaque extent (in other words, severity of subclinical atherosclerosis). Since distributions of plaque scores were not normally distributed, these values were natural-log transformed for analysis. For each outcome, we conducted two models: one included current or past smoking and all other substance use variables, and the other used cumulative years of smoking to replace current or past smoking variables. All models were performed stratified by HIV serostatus and adjusted for age, race, study center (Baltimore, Pittsburgh, Chicago, or Los Angeles), cohort status (enrolled pre- or post-2001), education (≥ college or not), and established CAD risk factors (use of hypertension or diabetes or lipid-lowering medications, systolic BP, fasting glucose, total cholesterol, and body mass index). The full, non-stratified models also included interaction terms to evaluate the shared effect of smoking and HIV infection on plaque presence and extent. In models evaluating HIV+ men alone, HIV clinical parameters (nadir CD4 count, detectable HIV RNA status, and history of AIDS-defining illness) were included. Multiple imputation was used to complete missing covariates for multiple regression models, with subsequent adjustment for the imputed data. For each outcome, the multiple imputation included all predictors and the outcome. Missing values were imputed five times based on the distribution of covariates using a Markov chain Monte Carlo (MCMC) method [34] assuming multivariate normality. Values for the following number of men were missing and imputed for multiple regression analyses: hypertension medications (14), systolic blood pressure (7), diabetes medications (16), fasting glucose (31), lipid medications (30), total cholesterol (4), body mass index (14), and current or past smoking (9). All statistical analyses were performed using SAS 9.3 (SAS Institute, Cary, NC). The statistical power of different comparisons, as different groups contain different numbers of participants, may have varied, however as this is a multiple regression analysis using observational data, formal power analyses were not performed, as is standard procedure.

## Results

There were 1005 men (621 HIV+ and 384 HIV-) who underwent non-contrast CT scanning to measure coronary artery calcium. Of these, 764 (453 HIV+ and 311 HIV-) had coronary CT angiograms. Participant characteristics are summarized on Table 1. Compared to HIV- men, HIV+ men were younger, more likely to be African American, and less likely to have college degree. HIV+ men had greater pack-years of smoking, were more likely to be current smokers, more likely to binge drink, and to use stimulants and inhaled nitrites.

**Table 1:** Participant Characteristics and Plaque/Stenosis Prevalence.

	Non-contrast CT		p-value	Non-contrast CT and CT-angiogram		p-value
	HIV+	HIV-		HIV+	HIV-	
	N = 621	N = 384		N = 453	N = 311	
**Age in years, Median (IQR)**	53(48–58)	55(50–62)	<0.001	52(47–57)	55(50–62)	<0.001
**Race**			<0.001			<0.001
**Caucasian (%)**	52.7	66.9		50.1	68.5	
**African American (%)**	34	24.7		34.7	23.8	
**Hispanic/Other (%)**	13.4	8.3		15.2	7.7	
**Education with college degree (%)**	43.2	59.9	<0.001	42.6	61.1	<0.001
**Systolic BP, mmHg, Median (IQR)**	126(115–137)	128(118–137)	0.25	126(115–136)	128(118–137)	0.095
**LDL cholesterol, mg/dL, Median (IQR)**	104(81–128)	112(91–137)	<0.001	104(82–134)	113(91–139)	0.001
**HDL cholesterol, mg/dL, Median (IQR)**	46(38–55)	52(43–61)	<0.001	45(37–55)	52(43–61)	<0.001
**Taking BP medication (%)**	35.9	31.2	0.132	32.1	29.8	0.51
**Taking DM medication (%)**	8.7	7.6	0.55	7.8	6.5	0.5
**Taking cholesterol medication (%)**	34.9	30.1	0.12	33.7	31.6	0.54
**HIV clinical factors**						
**Nadir CD4 (cells/μL), Median (IQR)**	248(144–336)	—		256(159–340)	—	
**History of AIDS (%)**	14.2	—		11	—	
**HIV RNA >50 copies/mL (%)**	17.9	—		19.6	—	
**Substance use prevalence**						
**Smoking status**			0.004			0.005
**Never (%)**	25.2	25.3		26.2	23.8	
**Former (%)**	43.8	52.9		43.5	54.7	
**Current (%)**	31.0	21.8		30.4	21.5	
**Pack years of smoking, Median (IQR)**	5.6(0–22.9)	1.8(0–21.5)	0.018	5.6(0–21.4)	3.1(0–21.9)	0.19
**Any ETOH use 1–14 drinks/week (%)**	71.7	78.1	0.026	72.8	81	0.005
**Any ETOH use >14 drinks/week (%)**	21.4	26.8	0.049	23	27.3	0.17
**Any binge drinking/week (%)**	31.2	25.5	0.052	33.3	26.4	0.04
**Any stimulant use (%)**	44.8	31.0	<0.001	47.7	30.9	<0.001
**Cumulative years of stimulant use, Median (IQR)**	3.4(0.6–17.4)	2.5(0.7–15.8)	0.666	3.7(0.7–17.3)	2.4(0.7–14.5)	0.294
**Any marijuana use (%)**	58.8	54.9	0.23	59.8	55.3	0.21
**Cumulative years of marijuana use, Median (IQR)**	3.7(0.7–29.5)	2.7(0.5–22.1)	0.321	3.9(0.6–31.2)	2.9(0.6–19.9)	0.615
**Any inhaled nitrite use (%)**	50.1	40.6	0.003	52.1	40.8	0.002
**Cumulative years of inhaled nitrite use, Median (IQR)**	2.5(0.5–11.4)	2(0.5–9.8)	0.581	2.6(0.5–13.4)	2.1(0.5–14.5)	0.418
**Any EDD use (%)**	48.8	45.6	0.32	48.3	44.1	0.24
**Cumulative years of EDD use, Median (IQR)**	3.1(1.1–6.3)	2.5(1–5.8)	0.198	2.9(1.1–6)	2.6(1–5)	0.383
**Plaque/Stenosis Prevalence (%)**						
**Prevalence of coronary artery calcium**	53.1	52.1	0.74	—	—	
**Prevalence of any plaque**	—	—		77.7	74.6	0.32
**Prevalence of calcified plaque**	—	—		34.9	39.9	0.16
**Prevalence of non-calcified plaque**	—	—		63.1	52.7	0.004
**Prevalence of mixed plaque**	—	—		34.7	31.8	0.47
**Prevalence of coronary artery stenosis >50%**	—	—		16.8	14.5	0.39

Among HIV+ men, current smoking was positively associated with the presence of coronary artery calcium, total plaque, calcified plaque and coronary artery stenosis >50% (OR [95% CI] 2.3 [1.3–3.9], 2.3 [1.1–4.7], 2.0 [1.1–3.9], 2.6 [1.1–6.0]) (Table 2), and with extent of coronary artery calcium (β [SE] 0.96 [0.27]) (Table 3). Former smoking was positively associated with presence of calcified plaque and stenosis (OR 2.2 [1.2–3.8], 2.2 [1.1–4.7]) (Table 2), and with extent of coronary artery calcium and total plaque (β 0.72 [0.23], β 0.24 [0.11]) (Table 3). Heavy alcohol use was positively associated with presence of stenosis (OR 4.7 [1.5–14.8]) (Table 2). Cumulative pack-years of smoking and EDD use were also significantly associated with multiple plaque measures, but with negligible effect sizes (Tables 2 and 3). No significant associations were apparent between plaque presence or extent and marijuana, stimulant or nitrite use among HIV+ men. There was an interaction of former smoking and HIV infection status with calcified plaque presence (p = 0.014) and coronary artery calcium extent (p = 0.009).

**Table 2:** Associations Between Substance Use and Presence of each Plaque Type in HIV+ Men.

Substance	Total Agatston Score >0	Total Plaque Score >0	Total Calcified Plaque Score >0	Total Non-calcified Plaque Score >0	Total Mixed Plaque Score >0	Stenosis >50%
	N = 621	N = 453	N = 453	N = 453	N = 453	N = 453
	OR (95% CI)	OR (95% CI)	OR (95% CI)	OR (95% CI)	OR (95% CI)	OR (95% CI)
**Current smoking**^**#**^	2.26** **(1.31–3.88)**	2.27* **(1.11–4.67)**	2.03* **(1.07–3.85)**	1.6 (0.88–2.93)	1.75 (0.93–3.29)	2.55* **(1.09–6.0)**
**Past smoking**^**#**^	1.59 (0.99–2.53)	1.63 (0.88–3.02)	2.17** **(1.24–3.78)**	1.22 (0.72–2.06)	1.46 (0.85–2.51)	2.22* **(1.05–4.7)**
**Cumulative pack-years**^**†**^	1.02** **(1.01–1.03)**	1.03** **(1.01–1.05)**	1.02** **(1.01–1.03)**	1.0 (0.99–1.02)	1.01* **(1.0–1.02)**	1.01 (1–1.03)
**Drinking 1–14 drinks/week**^**#**^	1.13 (0.73–1.76)	0.87 (0.47–1.62)	1.18 (0.71–1.97)	0.74 (0.45–1.22)	1.14 (0.69–1.89)	1.04 (0.54–2.0)
**Drinking >14 drinks/week**^**#**^	2.01 (0.75–5.4)	1.93 (0.36–10.23)	1.04 (0.37–2.92)	2.12 (0.64–7.05)	2.4 (0.87–6.59)	4.71** **(1.5–14.76)**
**Binge drinking**^**&**^	1.24 (0.52–2.95)	1.23 (0.35–4.27)	2.62 (0.95–7.23)	0.52 (0.18–1.47)	1.74 (0.65–4.69)	1.13 (0.31–4.08)
**Cumulative stimulant use**^**‡**^	1.0 (0.99–1.01)	1.0 (0.99–1.01)	0.99 (0.98–1.01)	0.52 (0.18–1.47)	1.0 (1.0–1.01)	1.0 (0.96–1.01)
**Cumulative marijuana use**^**‡**^	1.0 (1.0–1.01)	1.0 (1.0–1.01)	1.0 (1.0–1.004)	1.0 (0.99–1.01)	1.0 (1.0–1.004)	1.0 (1.0–1.004)
**Cumulative inhaled nitrite use**^**‡**^	1.0 (0.99–1.01)	1.0 (0.99–1.01)	1.0 (1.0–1.01)	1.0 (1.0–1.002)	1.0 (0.99–1.01)	1.0 (1.0–1.01)
**Cumulative EDD use**^**‡**^	1.01 (0.94–1.09)	0.95 (0.85–1.06)	1.01 (0.93–1.09)	1.0 (0.99–1.01)	1.06 (0.98–1.15)	1.05 (0.95–1.15)

*p≤ 0.05

**p≤ 0.01

^**#**^compared to no-use

^&^compared to non-binge drinking

†per pack-year (packs per day, per year)

‡per use-year (days of use per month, per year)

Model was adjusted for age, race, study center (Baltimore, Pittsburgh, Chicago, or Los Angeles), cohort status (enrolled pre- or post-2001), education (≥ college or not), and established CAD risk factors (use of hypertension or diabetes or lipid-lowering medications, systolic BP, fasting glucose, total cholesterol, and body mass index)

**Table 3:** Associations Between Substance Use and Extent of each Plaque Type in HIV+ Men.

Substance	Total Agatston Score	Total Plaque Score	Total Calcified Plaque Score	Total Non-calcified Plaque Score	Total Mixed Plaque Score
	N = 330	N = 352	N = 158	N = 286	N = 157
	β (SE)	β (SE)	β (SE)	β (SE)	β (SE)
**Current smoking**^**#**^	0.96** **(0.27)**	0.24 (0.13)	0.28 (0.2)	-0.07 (0.12)	0.13 (0.18)
**Past smoking**^**#**^	0.72** **(0.23)**	0.24* **(0.11)**	0.043 (0.17)	0.06 (0.11)	0.22 (0.16)
**Cumulative pack-years**^**†**^	0.01 (0.004)	0.01* **(0.002)**	0.01** **(0.003)**	-0.001 (0.002)	0.01 (0.003)
**Drinking 1–14 drinks/week**^**#**^	0.07 (0.21)	-0.02 (0.1)	-0.04 (0.15)	-0.01 (0.1)	0.06 (0.14)
**Drinking >14 drinks/week**^**#**^	0.44 (0.38)	0.35 (0.2)	0.34 (0.29)	0.34 (0.19)	-0.28 (0.26)
**Binge drinking**^**&**^	0.57 (0.4)	0.27 (0.22)	0.15 (0.28)	-0.03 (0.25)	0.26 (0.29)
**Cumulative stimulant use**^**‡**^	0.001 (0.01)	-0.002 (0.002)	0.001 (0.01)	-0.002 (0.002)	0.001 (0.003)
**Cumulative marijuana use**^**‡**^	-0.001 (0.001)	0 (0.001)	0 (0.001)	0 (0.001)	0 (0.001)
**Cumulative inhaled nitrite use**^**‡**^	-0.004 (0.003)	-0.001 (0.002)	-0.001 (0.002)	-0.001 (0.002)	0.001 (0.002)
**Cumulative EDD use**^**‡**^	0.03 (0.03)	0.02 (0.02)	0.06* (0.02)	0.001 (0.02)	-0.01 (0.02)

*p≤ 0.05

**p≤ 0.01

^**#**^compared to no-use

^&^compared to non-binge drinking

†per pack-year (packs per day, per year)

‡per use-year (days of use per month, per year)

Model was adjusted for age, race, study center (Baltimore, Pittsburgh, Chicago, or Los Angeles), cohort status (enrolled pre- or post-2001), education (≥ college or not), and established CAD risk factors (use of hypertension or diabetes or lipid-lowering medications, systolic BP, fasting glucose, total cholesterol, and body mass index)

Among HIV- men, current smoking was positively and significantly associated with calcified plaque extent only (β 0.49 [0.23]), however it is noteworthy that non-significant associations between current smoking and all plaque end-points, except non-calcified plaque, demonstrated large effect sizes similar in magnitude to those seen among HIV+ men (Tables 4 and 5). No associations were observed among former smokers. Cumulative pack-years of smoking was significantly associated with presence of coronary artery calcium, stenosis, and total plaque extent, while marijuana use was significantly associated with coronary artery calcium extent and stimulant use with total plaque presence, but all of these associations had negligible effect sizes (Tables 4 and 5). Moderate and heavy alcohol use were inversely associated with coronary artery calcium extent (β -0.69 [0.28], β -1.14 [0.51]), heavy alcohol use was inversely associated with calcified plaque extent (β -0.89 [0.27]) and binge drinking was positively associated with calcified plaque extent (β 0.85 [0.37]) (Table 5). No statistically significant associations between plaque and EDD or nitrite use were seen among HIV- men.

**Table 4:** Associations Between Substance Use and Presence of each Plaque Type in HIV- Men.

Substance	Total Agatston Score >0	Total Plaque Score >0	Total Calcified Plaque Score >0	Total Non-calcified Plaque Score >0	Total Mixed Plaque Score >0	Stenosis >50%
	N = 384	N = 311	N = 311	N = 311	N = 311	N = 311
	OR (95% CI)	OR (95% CI)	OR (95% CI)	OR (95% CI)	OR (95% CI)	OR (95% CI)
**Current smoking**^**#**^	1.85 (0.82–4.18)	1.76 (0.63–4.9)	2.04 (0.83–5.02)	0.84 (0.38,1.86)	1.79 (0.7–4.48)	2.55 (0.76–8.63)
**Past smoking**^**#**^	0.93 (0.51–1.71)	0.92 (0.43–1.95)	0.71 (0.36–1.38)	0.77 (0.43–1.39)	0.95 (0.47–1.92)	1.21 (0.48–3.08)
**Cumulative pack-years**^**†**^	1.02* (1.0–1.03)	1.01 (0.98–1.03)	1.01 (1.0–1.03)	0.99 (0.98–1)	1.01 (0.99–1.03)	1.02* (1.0–1.04)
**Drinking 1–14 drinks/week**^**#**^	1.18 (0.67–2.08)	1.24 (0.61–2.51)	0.96 (0.51–1.78)	0.88 (0.51–1.53)	0.79 (0.42–1.48)	0.59 (0.25–1.4)
**Drinking >14 drinks/week**^**#**^	0.83 (0.33–2.11)	2.55 (0.69–9.45)	1.09 (0.38–3.11)	1.58 (0.61–4.13)	0.46 (0.14–1.46)	0.68 (0.16–2.89)
**Binge drinking**^**&**^	0.44 (0.13–1.46)	0.48 (0.13–1.76)	0.33 (0.09–1.2)	0.61 (0.21–1.71)	0.33 (0.08–1.46)	2.23 (0.49–10.1)
**Cumulative stimulant use**^**‡**^	1.02 (1.0–1.04)	1.04* **(1.001–1.08)**	1.02 (1.0–1.04)	1.0 (1.0–1.02)	1.01 (0.99–1.03)	0.99 (0.97–1.02)
**Cumulative marijuana use**^**‡**^	1.0 (1.0–1.0)	1.0 (0.99–1.01)	1.0 (0.99–1.004)	1.0 (1.0–1.01)	1.0 (1.0–1.01)	1.0 (1.0–1.01)
**Cumulative inhaled nitrite use**^**‡**^	1.01 (1.0–1.03)	1.0 (0.98–1.02)	1.0 (0.98–1.02)	1.01 (0.99–1.02)	1.0 (1.0–1.02)	1.0 (0.98–1.02)
**Cumulative EDD use**^**‡**^	1.03 (0.93–1.14)	0.93 (0.81–1.06)	0.99 (0.89–1.11)	0.96 (0.87–1.07)	1.03 (0.92–1.16)	0.87 (0.73–1.04)

*p≤ 0.05

^**#**^compared to no-use

^&^compared to non-binge drinking

†per pack-year (packs per day, per year)

‡per use-year (days of use per month, per year)

Model was adjusted for age, race, study center (Baltimore, Pittsburgh, Chicago, or Los Angeles), cohort status (enrolled pre- or post-2001), education (≥ college or not), and established CAD risk factors (use of hypertension or diabetes or lipid-lowering medications, systolic BP, fasting glucose, total cholesterol, and body mass index)

**Table 5:** Associations Between Substance Use and Extent of each Plaque Type in HIV- Men.

Substance	Total Agatston Score	Total Plaque Score	Total Calcified Plaque Score	Total Non-calcified Plaque Score	Total Mixed Plaque Score
	N = 200	N = 232	N = 124	N = 164	N = 99
	β (SE)	β (SE)	β (SE)	β (SE)	β (SE)
**Current smoking**^**#**^	0.61 (0.42)	0.28 (0.17)	0.49* **(0.23)**	-0.08 (0.16)	-0.07 (0.31)
**Past smoking**^**#**^	-0.36 (0.31)	-0.02 (0.131)	-0.14 (0.18)	0.11 (0.12)	-0.26 (0.23)
**Cumulative pack-years**^**†**^	0.01 (0.01)	0.01* **(0)**	0.01 (0.004)	0.004 (0.003)	0 (0.004)
**Drinking 1–14 drinks/week**^**#**^	-0.69* **(0.28)**	-0.16 (0.12)	-0.22 (0.15)	0.03 (0.11)	-0.34 (0.2)
**Drinking >14 drinks/week**^**#**^	-1.14* **(0.51)**	-0.32 (0.2)	-0.89** **(0.27)**	-0.004 (0.18)	-0.47 (0.38)
**Binge drinking**^**&**^	0.70 (0.66)	0.28 (0.24)	0.85* **(0.37)**	0.36 (0.23)	0.29 (0.53)
**Cumulative stimulant use**^**‡**^	0 (0.01)	0 (0.003)	-0.01 (0.004)	-0.003 (0.004)	0.01 (0.01)
**Cumulative marijuana use**^**‡**^	0.01* **(0.002)**	0 (0.001)	0 (0.001)	0.001 (0.001)	0 (0.002)
**Cumulative inhaled nitrite use**^**‡**^	-0.01 (0.01)	0 (0.003)	0 (0.01)	0 (0.003)	-0.002 (0.01)
**Cumulative EDD use**^**‡**^	0.08 (0.05)	-0.01 (0.02)	0.03 (0.03)	-0.04 (0.02)	-0.01 (0.04)

*p≤ 0.05

**p≤ 0.01

^**#**^compared to no-use

^&^compared to non-binge drinking

†per pack-year (packs per day, per year)

‡per use-year (days of use per month, per year)

Model was adjusted for age, race, study center (Baltimore, Pittsburgh, Chicago, or Los Angeles), cohort status (enrolled pre- or post-2001), education (≥ college or not), and established CAD risk factors (use of hypertension or diabetes or lipid-lowering medications, systolic BP, fasting glucose, total cholesterol, and body mass index)

## Discussion

In this well-characterized group of HIV+ and HIV- men who underwent CT evaluation of coronary plaque, cigarette smoking was positively associated with more measures of subclinical atherosclerosis presence and extent with large effect sizes among HIV+ men than among HIV- men. Among HIV+ men, current smoking was associated with presence of coronary artery calcium, any plaque, calcified plaque, coronary stenosis >50%, and extent of coronary artery calcium. Past smoking was associated with presence of calcified plaque and stenosis, as well as extent of coronary artery calcium and total plaque. There was a positive interaction between HIV infection and past smoking on calcified plaque presence and coronary artery calcium extent, suggesting a unique synergistic effect on these plaque endpoints. Among HIV- men, current smoking was associated with calcified plaque extent, though no other significant interactions with a large magnitude of effect were evident. Non-significant associations, but with large effect sizes, were noted between current smoking and presence of coronary artery calcium, total plaque, calcified plaque, mixed plaque and stenosis >50% among HIV- men; this indicates a potential non-significant trend of positive association between current smoking and each plaque type except for non-calcified plaque, however the non-statistical significance might also be due to smaller sample size than the HIV+ group. Nonetheless, we did observe a significant positive interaction between HIV infection and past smoking on calcified plaque presence and coronary artery calcium extent, as stated above. While prior cross-sectional studies have demonstrated associations between coronary artery calcium and HIV infection [5,11], our observed differential associations of smoking with subclinical atherosclerosis by HIV serostatus are novel.

The prevalence of tobacco smoking among HIV+ persons is higher than in the general population [35]. Increased risk for CVD already exists among HIV+ persons [2], and HIV+ smokers have been estimated to carry twice the risk of a major cardiovascular event compared to HIV+ nonsmokers [36]. HIV infection and smoking independently trigger an inflammatory cascade in which endothelial activation, smooth muscle and fibroblast proliferation, and eventual endothelial dysfunction and plaque deposition take place [8,37], possibly accounting for this increased CVD risk. Variations in calcium composition among plaque can affect the clinical course of atherosclerosis; calcified plaque likely represents more long-standing, stable plaque, while non-calcified plaque may be newer plaque that is more prone to rupture [13,38]. Our group has recently demonstrated that HIV-infection is associated with a greater prevalence and extent of non-calcified plaque [12]. In the current study, however, we observed significant associations of smoking with calcified plaque prevalence and extent yet no significant associations with non-calcified plaque presence or extent among HIV+ men. Tobacco use may exert a modifying effect upon plaque among HIV+ men, predisposing plaque to calcium deposition, or selectively increasing the likelihood of calcified plaque formation. Furthermore, while the MACS cardiovascular study did not identify a significant association between HIV infection and stenosis >50% after adjusting for CVD risk factors including pack-years of smoking [12], our current findings suggest that current or past smoking among HIV+ men is significantly associated with stenosis >50%. Our more numerous observed associations between smoking and plaque presence and extent among HIV+ men support a hypothesis that HIV infection and tobacco use exert a synergistic effect in promoting atherosclerosis development.

Our findings underscore the importance of effective smoking cessation strategies targeting HIV+ persons to decrease CVD burden. Among HIV+ smokers, mostly small clinic-based randomized control trials of intensive counseling, group therapy, and combined nicotine replacement therapy have been reported, demonstrating inconsistent cessation rates and no follow-up beyond 6 months [39–42]. A recent randomized trial demonstrated higher abstinence rates at 48 weeks associated with varenicline use compared to placebo (17.6% vs 7.2%, respectively) [43], suggesting effectiveness of this smoking cessation strategy for HIV+ persons. Smoking cessation strategies have been shown to be less effective among persons using other recreational drugs [44,45], and HIV+ persons tend to use recreational drugs at higher rates than the general population, especially if they are smokers [21,22,44]. Thus, polysubstance use among HIV+ persons may be a significant barrier to quitting smoking.

Moderate (and even heavy) alcohol use has been associated with a protective effect against fatal and non-fatal cardiovascular events in the general population [46–50]. However, alcohol may also be pro-atherosclerotic with heavy use, causing oxidative damage, smooth muscle proliferation and impairment of endothelial repair mechanisms [51]. Increased coronary artery calcium prevalence has been observed in association with increasing self-reported alcohol use in the general population [52]. Our findings support a role for low/moderate and even heavy alcohol in the protection from coronary artery calcium and calcified plaque progression among HIV- men that was not observed among HIV+ men. Mechanisms accounting for these differential associations by HIV serostatus are unclear. Since alcohol consumption has been associated with lower levels of inflammation in HIV- adults without CVD [53], HIV infection may sufficiently alter the coronary vascular endothelial milieu through enhanced inflammation and immune activation to offset protective effects of alcohol use and/or to enhance alcohol’s pro-atherosclerotic effects. While no conclusions regarding the effects of low/moderate alcohol use upon coronary plaque formation among HIV+ men can be drawn from this study, our findings suggest that among HIV+ men, heavy alcohol consumption may be harmful. It is important to note that heavy drinking and binge drinking are not mutually inclusive (several subjects, for instance, drank >5 drinks in one episode and were thus classified as binge drinkers, yet overall consumed <14 drinks/week). The episodic and inconstant nature of binge drinking therefore likely contributes to inter-group variability in alcohol exposure between the HIV+ and HIV- binge drinkers, and heavy drinking and binge drinking in these and other analyses should be interpreted separately.

Use of other recreational drugs was not clearly associated with subclinical atherosclerosis. Only erectile dysfunction drug use (in HIV+ men), stimulant and marijuana use (in HIV- men) were positively associated with coronary artery plaque presence or extent, all with very small magnitudes of effect. It is possible that associations were diminished by coincident tobacco smoking, which was prevalent in both groups. Other than cocaine, none of the other recreational drugs we studied are known to be directly pro-atherosclerotic in the general population. Nonetheless, other recreational substance use may contribute to CVD risk indirectly by decreasing efficacy of smoking cessation strategies.

This study has several limitations. Approximately 75% of all participants were current or former smokers; the pro-atherosclerotic effects of smoking may have overshadowed effects of other substance use in coronary plaque development, despite analytic adjustment for smoking. This may be true for use of smoked and inhaled cocaine use; because such use was relatively rare in our cohort, our analysis may have been underpowered to see this effect. The differential associations with smoking and plaque endpoints between the HIV- and HIV+ groups may have also been influenced by the statistical power of the study. The smaller sample size of the HIV- group may have contributed to the non-significance of many associations despite having large effect sizes. Thus, there may be a trend of positive association between current smoking and all plaque types (except for non-calcified plaque) among HIV- men, the significance of which we were unable to observe, suggesting differences in plaque end-points among HIV+ and HIV- current smokers are not definitive in this study. Further, the larger sample size of the HIV+ group, compounded by the multiplicity of variables in the analysis, may have led to the significant associations we observed with very small effect sizes in this group. As such, true differences in subclinical atherosclerosis between the groups due to smoking may have been overestimated in our study. Furthermore, the self-reported nature of our data, possibly leading to underreporting, and the inclusion of men only are limitations. As our substance use data relied on the standardized questionnaire answer choices, we were unable to determine more precise frequencies of drug use and dose responses. Despite their diversity, MACS participants may not be generalizable to other MSM in the United States. Nevertheless, our study involved a large sample size, prospectively-collected data over 10 years rather than cross-sectional, detailed characterization of coronary artery plaque and stenosis, and an HIV- MSM control group that was similar in most respects to the HIV+ participants, which are all factors that minimize the likelihood of confounding and selection bias. It is important to note the exploratory quality of this observational study, as we are unable to derive clinical significance of these associations we report. Consequently, longitudinal studies evaluating these plaque end-points in the setting of substance use are needed to more definitely assess differences of substance use and subclinical atherosclerosis associations between HIV+ and HIV- men.

## Conclusion

HIV+ smokers are particularly vulnerable to CVD, which may reflect independent contributions of both HIV infection and smoking to inflammatory processes injurious to vascular endothelium. We observed a possible protective effect of low/moderate and heavy alcohol use from coronary artery calcium and calcified plaque progression in HIV- men but not in HIV+ men. While we speculate that the chronic inflammatory effects of HIV infection may offset such cardio-protective effects of alcohol use, further study is needed. Despite the known detrimental health consequences of recreational drug use, we did not find conclusive significant associations with subclinical atherosclerosis; any associations may have been less apparent because of the potential co-mingling with tobacco smoking. Nevertheless, recreational drug use may contribute to CVD burden indirectly by making smoking cessation more difficult. Collectively, our findings reinforce the need for effective smoking cessation strategies as a part of CVD prevention, and suggest that this is at least as important, if not more important, an undertaking for HIV+ smokers.

## Appendix

In this study, exposure to recreational drugs was assumed to be cumulative over the last 10 years (20 visits) before CT-scanning. That is, we assumed that the effect of drug use on the outcomes depended not only on current use, but also on previous use, but only for a period corresponding to 10 years. This seemed a reasonable compromise between the two extremes of using only current exposure and using a cumulative measure like pack-years of smoking that includes all exposures, no matter how far in the past. Cumulative recreational drug use was calculated for each visit as average number of reported days used per month for a one year period for stimulants (smoked or inhaled cocaine, methamphetamines, and ecstasy), marijuana, inhaled nitrites, and erectile dysfunction drugs (EDD; phosphodiesterase inhibitors). This involved using a measure of quantity or frequency in a given time interval depending on the wording of the particular question (the weight), during a given period, typically the approximately six months between visits, normalized to a yearly basis. We constructed a unit of cumulative use of recreational drugs called use-years (similar to pack-years, the standard measurement of cumulative smoking); use-years for recreational drugs were standardized to monthly use (as opposed to daily use with tobacco smoking). Thus, use-years were characterized as use of drug per month, per year. For the drug use questions, the weights were daily use: weight = 30.5 (times per month); weekly use: weight = 4.36; monthly use: weight = 1; less often: weight = 0.33. We then computed cumulative self-reported drug use over the 10-year period (20 visits) before CT-scan by summing the previous yearly averages at the 20 visits, which proceeded longitudinally across the 10-year study period. Similarly, additional primary exposure variables were measured as cumulative amounts per year. This also involved using a measure of quantity or frequency in a given time interval, depending on the wording of the particular question, normalized to a yearly basis. For example, if an individual smoked > = 1 but < 2 packs per day in the six months preceding a given visit, then his smoking pack-years exposure for that visit was calculated as 1.5 (the average of 1 and 2) packs x 0.5 years = 0.75 pack years. Current exposure was calculated at each visit and cumulated over visits.
